# New insights into the role of ribonuclease P protein subunit p30 from tumor to internal reference

**DOI:** 10.3389/fonc.2022.1018279

**Published:** 2022-10-13

**Authors:** Junchao Wu, Sijie Yu, Yalan Wang, Jie Zhu, Zhenhua Zhang

**Affiliations:** ^1^ Institute of Clinical Virology, Department of Infectious Diseases, The Second Hospital of Anhui Medical University, Hefei, China; ^2^ Department of Clinical Medicine, Anhui Medical University, Hefei, China

**Keywords:** RPP30, protein structure, tumor, internal reference gene, PCR diagnosis

## Abstract

Ribonuclease P protein subunit p30 (*RPP30*) is a highly conserved housekeeping gene that exists in many species and tissues throughout the three life kingdoms (archaea, bacteria, and eukaryotes). *RPP30* is closely related to a few types of tumors in human diseases but has a very stable transcription level in most cases. Based on this feature, increasing number of studies have used *RPP30* as an internal reference gene. Here, the structure and basic functions of *RPP30* are summarized and the likely relationship between *RPP30* and various diseases in plants and human is outlined. Finally, the current application of *RPP30* as an internal reference gene and its advantages over traditional internal reference genes are reviewed. *RPP30* characteristics suggest that it has a good prospect of being selected as an internal reference; more work is needed to develop this research avenue.

## Introduction

The ribonuclease P protein subunit P30 (*RPP30*) gene is included in the National Center for Biotechnology Information (NCBI ID#10556) database. *RPP30* has been shown to be highly conserved in gene pool data, and many studies have shown that there are 16 homologous genes of *RPP30* contained in many species from the three life kingdoms (archaea, bacteria, and eukaryotes) (1, 2). As a housekeeping gene, the protein encoded by *RPP30* is one of shared protein subunits of ribonuclease P (RNase P) and ribonuclease MRP (RMRP), which are widely expressed in various tissues and participate in many life processes of microscopic and macroscopic organisms. It should be noted that as a protein subunit, detection of *RPP30* in different tissues is not uniform and stable, possibly because of the complex modification process after translation (3–6).

In this review, the diseases associated with *RPP30* and the factors that may influence its expression are introduced for reference in further studies and in quality control. Abnormal gene expression or mutation studies have made some progress with regard to botanical diseases (5–7). At present, studies on human diseases mainly involve tumors, but only a few of them have demonstrated *RPP30* overexpression (8). In addition, *RPP30* is associated with glioblastoma (GBM) pathogenesis and low bone mineral density (LBMD) (9)

Reports that *RPP30* expression level is affected by other factors are very limited, such as aging (10). Given the relatively high and stable ribose nucleic acid (RNA) expression of *RPP30* in human tissues, increasing studies have recently used *RPP30* as an internal reference gene in reverse transcription-polymerase chain reaction (RT-PCR) protocols. Thus, the use of *RPP30* as an internal reference gene for many applications, including detection of pathogens, calculation of the number of tumor cells, diagnosis of tumors, and some childhood diseases are discussed. In particular, the application of this gene in nucleic acid detection of SARS-CoV-2 demonstrates its great value as an internal reference (11).

Finally, the advantages of *RPP30* over conventional reference genes, such as *β-actin* and glyceraldehyde-3-phosphate dehydrogenase (*GAPDH*) are discussed. Ideal reference genes should be stably expressed in different tissues and different life cycles. With increased research and more applications, the expression of *β-actin* and *GAPDH* has been observed to be related to physiological/pathological states, experimental conditions, and tissue type (12–14). In contrast, changes in *RPP30* expression seem less likely to be reported in the many conditions described above. These data suggest *RPP30* may be used as an internal reference gene in further studies. Of course, the reliability of *RPP30* as an internal reference is required to be verified by more comprehensive experiments.

## Gene and protein structure

**Figure 1:**
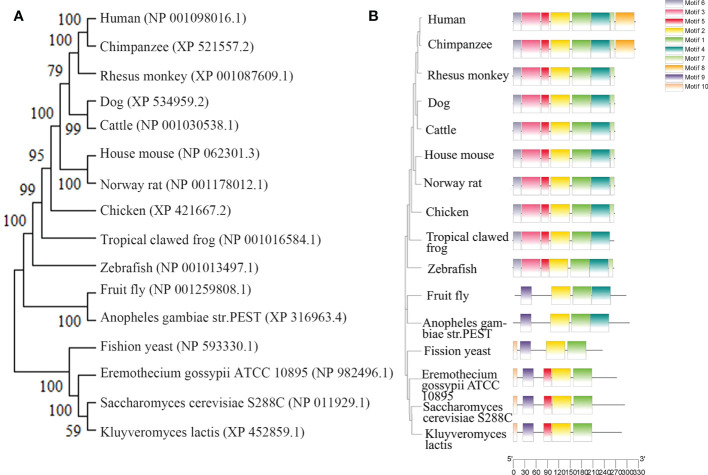
Homology of ribonuclease P protein (RPP) in various species. **(A)** Homologous evolutionary tree, **(B)** Human *RPP30* protein subunit, and homologous protein subunits of other organisms.

The highly conserved *RPP30* genome sequence is located on human chromosome 10 (10Q23.31) at 90,871,974–90,908,556 and is 36,582 nucleotides in length, with 14 exons (https://www.ncbi.nlm.nih.gov/gene/). There are 16 homologous genes in primates, canine, bovine, Rodentia, Amphibia, Drosophila, Arthropoda, and Saccharomycetes. These data are obtained from NCBI (ID#10556). The highly conserved sequence and other characteristics of *RPP30* are illustrated in a gene evolutionary tree in **Figure 1A**.

Human nuclear RNase P consists of 1 RNA subunit H1 and 10 conserved proteins, and the complex has a slender conformation similar to the overall shape of yeast RNase P shown by cryo-negative staining electron microscopy (2). The human RNase P protein consists of a single protein, Pop1, and three subcomplexes, which include the RPP20-RPP25 heterodimer, Pop5-RPP14-(RPP30)2-RPP40 heteropentamer, and RPP21-RPP29-RPP38 heterotrimer (2). The proteins are tightly attached to each other, forming a structure similar to a right-handed clip with three modules: finger, palm, and wrist. The POP5-RPP14-(RPP30)2-RPP40 heteropentamer becomes the palm module of the protein clamp. Two copies of the RPP30 molecule bind to the central POP5-RPP14 from opposite sides, forming a typical trisose phosphate isomerase (TIM) barrel fold. The molecule of RPP30 that interacts with RPP40 is called RPP30B, and the other molecule is called RPP30A. The secondary and tertiary structures of RPP30 have not been analyzed at this stage (2).

The RPP30 protein subunit is homologous to the RNase P protein subunit of archaea and other eukaryotes, as shown in **Figure 1B**. By comparing the amino acid sequence of homologous genes of *RPP30*, the domain and conserved site of the RPP30 protein may be identified, which will illustrate how conserved this protein is.

## Main functions of *RPP30*

### Life processes

*RPP30* mainly functions in catalysis, nuclear localization, assembly, and/or regulation of holoenzyme activity (3). The GeneCards (https://www.genecards.org/) and Gene Ontology (GO; http://geneontology.org/) databases were searched for the *RPP30* gene to identify its basic functions. The gene’s molecular functions include binding proteins, catalyzing reactions, and so on (15). In archaea, two *RPP30* copies bind with ribonuclease P/MRP protein subunit Pop5 dimers to form the Pop5•RPP30 heterodimer. The Pop5•RPP30 heterodimer is anchored on the catalytic domain of RNase P RNA(RPR), which is necessary for pre-tRNA cleavage (16). Cellular components encoded by the gene include RNase P complex, RNase MRP complex, and the multimeric ribonuclease P complex. Biological processes mediated by the gene include rRNA and tRNA processing (15). *RPP30* gene encodes a type of ribonuclease that achieves RNaseP RNA binding activity, contributes to ribonuclease P activity, and participates in the removal of tRNA5′- precursor, as well as the formation of polynuclease P complex, and ribonuclease MRP complex, which is necessary for the gene transcription of RNA polymerase III (17). *RPP30* also facilitates immunity in rice and reproduction in *Arabidopsis* and *Drosophila* (5–7). Therefore, as the most conserved gene in various types of organisms, *RPP30* is also involved in the most basic life processes, which drive almost all life functions and activities.

### A common subunit joining RNase P and RMRP

RNase P and RMRP are both small nucleolar ribonucleoprotein complexes (snoRNPs) that are classified into three major classes (box H/ACA snoRNPs, box C/D snoRNPs, RNase P and RnaseMRP) (18). RMRP has only been found in eukaryotes, located mostly in the nucleolus, and has many functions, including cleaving the pre-rRNA at site A3 *in vivo* and *in vitro* to mature the 5′ end of the 5.8S rRNA, cleaving an RNA transcript to generate RNA primers for mitochondrial DNA duplication, cleaving the B-type cyclin, Clb2, mRNA, recognizing and cutting pre-tRNA, and is required to turn over cell cycle mRNA (19–21). RNase P, located both in the nucleoplasm and nucleolus, is necessary for Mg^2+^ dependent 5′ maturation of tRNAs in archaeal, bacterial, and eukaryotic kingdoms (22). RNase P may also act in the stress response and be a transcription factor that regulates polymerase I and III (23). RNAs containing N6 methyladenosine (m6A), 4.5S pre-rRNA, operon mRNAs, box C/D small nucleolar RNAs that reassemble tRNAs are also substrates of RNase P (15, 24–30). RNase P and RMRP have similar functional and structural characteristics (18, 31). These two enzymes share at least ten protein subunits, including RPP14, RPP20, RPP21, RPP25, RPP29, RPP30, RPP38, RPP40, Pop1, and Pop5 (4, 15). *RPP30*, with a highly conserved amino acid sequence, has an important role in joining the RNase P and RMRP complexes (32). RPP30, as one of the common subunits between the RNase P and RMRP complexes, contributes to an increased number of RNA substrates and atypical functions of eukaryotes (4, 33). RNase P H1 and RMRP RNAs may crosstalk with miRNAs that are related to stability and translation of mRNAs (34). Stolc and Altman reveal that reduction RPP1 (homologous to human *RPP30*) in *S. cerevisiae* causes disruptions in both RNase P and RMRP by inhibiting correct cleavage of the internal transcribed spacer I of rRNA surrounding the A3 site (35).

### A cofactor acting with the RNA subunit and other protein subunits of RNase P or RMRP

Although RNase P RNA (RPR) is suggested to have activity *in vitro*, its activity *in vivo* requires protein cofactors (36). In 2006, Welting et al. used glycerol gradient sedimentation and coimmunoprecipitation to determine that *RPP30* is related to the RNA subunit of *RNase P* and *RMRP* (18). UV–crosslinking studies also show that *RPP30* interacts directly with *H1* RNA, an RNA subunit of *RNase P* (37, 38). Isothermal titration calorimetry has been used to explore interactions among the protein subunits of RNase P and RMRP (22, 39, 40). In archaea, bacteria, and yeast, RPP30/RPP30 paired with Pop5/Pop5, may be functionally reconstituted with the phylogenetically-conserved core catalytic domain (C domain) of the RNA subunit to promote the assembly of RNase P providing substrate RNA binding sites and activating the RNA subunit (probably by RNA annealing and strand displacement (41) and stabilize ionic interactions with the RNA subunit or the substrate pre-tRNA at a relatively lower salt concentration (1, 22, 42–44). In the hyperthermophilic archaeon *Pyrococcushorikoshii*, PhoRPP30 is homologous to human RPP30 and acts as a molecular chaperone of PhoPop5, which recognizes the stem-loop containing the P3 helix in PhopRNA (45). RPP30-Pop5 is a tight heterotetrameric complex that increases the affinity of the holoenzyme for Mg^2+^ and protects the RNase P M1 RNA’s C domain from RNase T1 cleavage, especially near conserved nucleotides of RNase P in archaea whose RNase P protein is homologous to eukaryotic counterparts (36, 46–48). The RPP30-Pop5 complex also increases the RPR cleavage rate of pre-tRNA and may be activated by the RPP21-RPP29 complex reflecting indirect effects (36). In *Dictyosteliumdiscoideum*, RPP30 adopts a TIM-barrel fold that stabilizes the structure and enhances the affinity of pre-tRNA of RNase P to promote the formation of a native fold (46, 49, 50). In humans, RPP30 interacts with RPP14, RPP40, RPP20, RPP21, Pop1, RPP29, 4pp38, and RPP30 itself (15, 37, 51). Moreover, Stolc and Altman have shown that the *RPP30* and *RPP38* cDNA code for proteins related to catalytic complexes of RNase P from HeLa cells (35). Additionally, *RPP30* may interact with other RNAs; as an important subunit of RNase P, RPP30 may be involved in the cleavage of hepatitis C virus RNA (52).

### Regulation of biological procedures in other species

In *Arabidopsis*, the *RPP30* domain is present from 98–248 amino acids in gametophyte defective 1 (*GAF1*), which is important in female gametophyte development and male competence and has a universal contribution to plant development (5). In *Drosophila*, *RPP30* is necessary for female oogenesis because of its relationship with tRNA processing, DNA replication, and piRNA transcription (7). *RPP30* also positively regulates rice immunity by interacting with histone deacetylase 701 (*HDT701*, *RPP30* may be a substrate of *HDT701*), which functions in suppressing innate immunity in rice and may upregulate expression of defense genes (6).

Although many functions of *RPP30* have recently been identified, the specific role of *RPP30* in basic life processes requires further research.

## Relationship between *RPP30* and disease

RNase P and RMRP play an important role in RNA or non-RNA processing that are universal programs closely related to many life activities. As an important subunit, the mutation and abnormal expression of *RPP30* leads to many diseases.

### *RPP30* mutations and reproductive diseases

In *Arabidopsis*, *GAF1* mutations result in decreased *RPP30* levels that induce defects in mitosis during female gametophyte development, arrest embryo sacs at stages FG1–FG7 and also cause defects in male competence (5). In *Drosophila*, an isolated mutation that inserts the P-element P(lacW)k01901 into *RPP30* leads to complete sterility in females (7, 49). The pathogenic mechanisms that have been uncovered include a mutation in *RPP30* that arrests oogenesis by decreasing tRNA processing, which leads to transcription-replication conflicts (7). This includes decreases in transposon expression, accumulation of the polymerase III subunit Brf, and the collapse of Proliferating Cell Nuclear Antigen (PCNA), which increases DNA replication stress and gene defense by small RNAs and activates several DNA duplication checkpoint proteins, including p53, claspin, and checkpoint kinase 2 that decrease piRNA transcription and piRNAclusterpopulations (7). piRNAs are native defenders of germline cell genomes whose mature structure called a “nuage” surrounds the nurse cells that provide nutrients to oocytes (53). Additionally, downregulation of piRNA levels leads to derepression of transposable elements and activates DNA checkpoints to promote positive feedback of defective oogenesis (7, 53–56).

### A factor that protects plants against pathogens

Li et al. have identified OsRPP30, a cellular protein that may regulate the biological function of rice *HDT701* (6). *HDT701* negatively regulates defense mechanisms in rice by increasing histone H4 deacetylation and increasing the sensitivity to *Magnaporthe grisea* and *Xanthomonas oryzae*pv.*oryzae* (57). When rice is infected with *Pyriculariaoryzae* (syn. *Magnaportheoryzae*), *RPP30* expression increases, which activates the transcription of defense genes (6). The overexpression of OsRPP30 in genetically modified rice increases expression of defense genome and the production of reactive oxygen species, resulting in resistance to *Magnaporthe grisea* and *Xanthomonas oryzae*. OsRPP30 is located at the top of the immune pathway triggered by HDT701-mediated pathogen-associated molecular patterns, which may overcome the negative effects of HTD701 and provide a new direction for the cultivation of pathogen-resistant food in the future (6).

### Anti-RPP30 antibody and autoimmune diseases in humans

Anti-Th/To is one of the rarer antinuclear antibodies identified in patients with systemic sclerosis (SSc) and is composed of hPOP1, RPP25, RPP30, and RPP40 (58, 59). Researchers refer to “anti-Th” and “anti-To” in the cases of RNase MRP and RNase P, respectively (60, 61). Recombinant RPP30 and RPP38 cross-react with anti-Th/To antibodies of patients afflicted with SSc (3, 32). In addition, people with positive anti-RPP30 antibodies have a lower risk of tendon friction rubs and cancer, but more likely to have severe lung diseases and pulmonary hypertension (59, 62). However, the positivity of anti-RPP30 antibodies only represents the antigenicity of RPP30 protein, and does not suggest the existence of abnormal expression or a *RPP30* gene defect, which requires further research.

### RPP30 and human tumors

**Figure 2:**
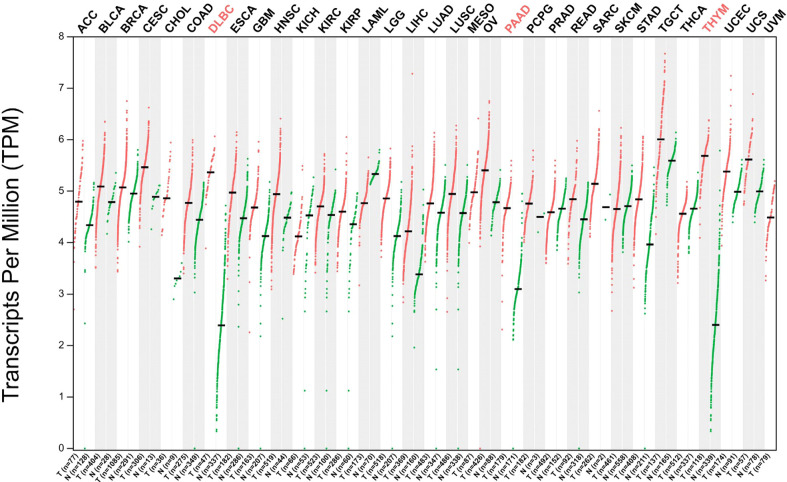
Gene expression profile of ribonuclease P protein subunit p30 (*RPP30*) in all tumor samples and paired normal tissues. Red words: significant difference; Black words: No significant difference. Abbreviations: ACC: Adrenocortical carcinoma, Bladder Urothelial Carcinoma, Breast invasive carcinoma, Cervical squamous cell carcinoma and endocervical adenocarcinoma, Cholangio carcinoma, Colon adenocarcinoma, Diffuse Large B-cell Lymphoma, Esophageal carcinoma, Glioblastoma multiforme, Head and Neck squamous cell carcinoma, Kidney Chromophobe, Kidney renal clear cell carcinoma, Kidney renal papillary cell carcinoma, Acute Myeloid Leukemia, Brain Lower Grade Glioma, Liver hepatocellular carcinoma, Lung adenocarcinoma, Lung squamous cell carcinoma, Mesothelioma, Ovarian serous cystadenocarcinoma, Pancreatic adenocarcinoma, Pheochromocytoma and Paraganglioma, Prostate adenocarcinoma, Rectum adenocarcinoma, Sarcoma, Skin Cutaneous Melanoma, Stomach adenocarcinoma, Stomach and Esophageal carcinoma, Testicular Germ Cell Tumors, Thyroid carcinoma, Thymoma, Uterine Corpus Endometrial Carcinoma, Uterine Carcinosarcoma, Uveal Melanoma, in turn.

**Figure 3:**
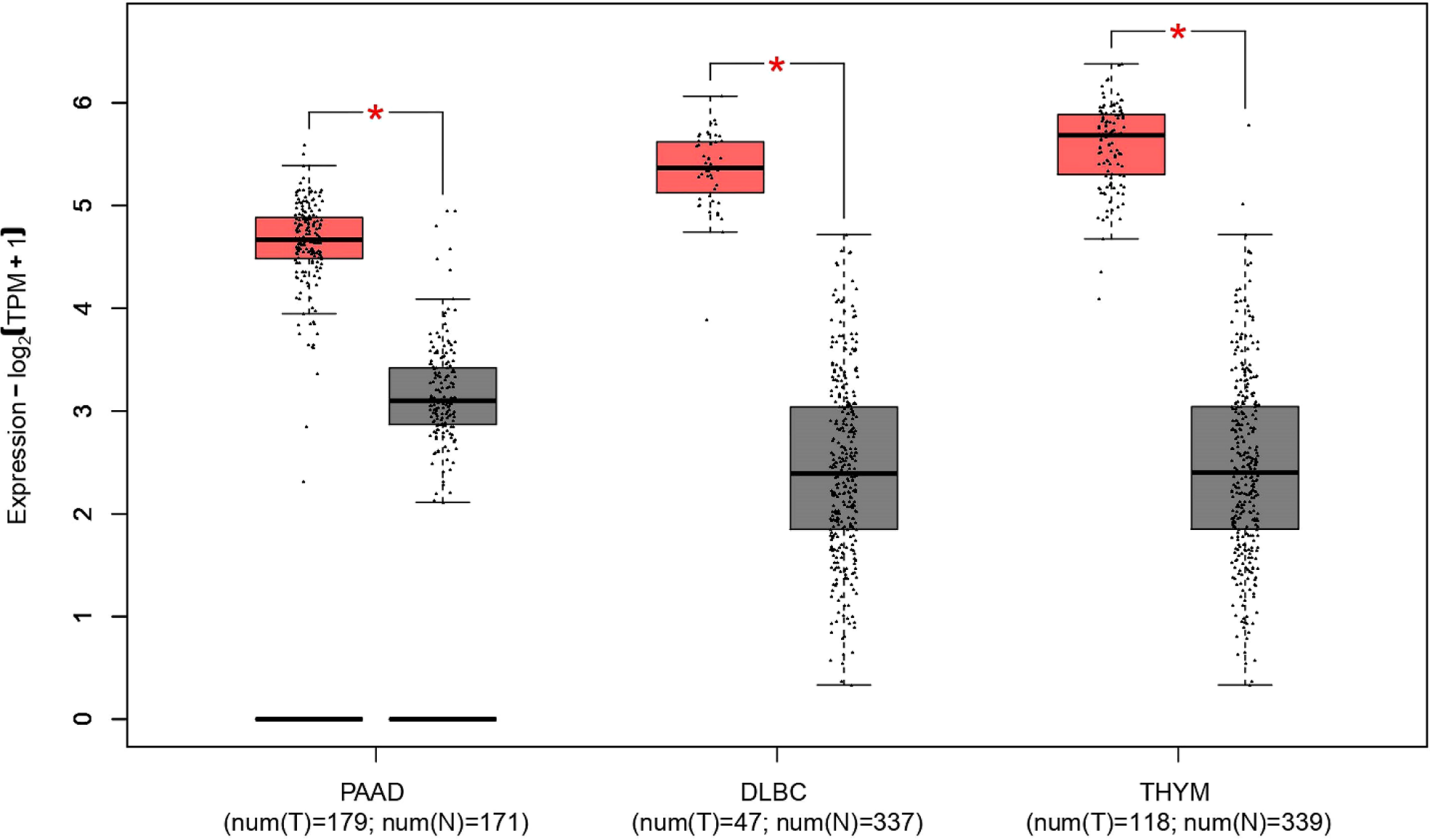
Differences in ribonuclease P protein subunit p30 (*RPP30*) expression between DLBC, PAAD, THYM, and normal tissues. *Significant difference between tumor and normal tissues; DLBC, Diffuse large B-cell lymphoma; PAAD, Pancreatic adenocarcinoma; THYM, Thymoma. Red represents tumor group, gray represents normal group.

The nucleophosmin (*NPM1*) gene, located at human chromosome 5Q35, contains 12 exons and encodes a multifunctional shuttling protein that shuttles between the nucleolus and cytoplasm. *NPM1* mutations happen in approximately one-third of acute myeloid leukemias (AMLs) (63). Martelli et al. have shown that the NPM1/RPP30 complex serves as one of three *NPM1* rearrangements that have been found and analyzed in 13,979 AML samples (64). In patients with AML that have a *NPM1* rearrangement, *RPP30* is rearranged with *NPM1* at exon 11, whereas the rearrangement of *NPM1* with *RPP30* is at the end of exon 9 (64). These data indicate *RPP30* may help detect AML and monitor NPM1-mutated AML. A new study found that *RPP30* may be a transcriptional regulator in glioblastoma (GBM) and the decreased *RPP30* expression in elderly people could be a risk factor for GBM (10). This study showed that *RPP30* was related to RNA and post-transcriptional modification in non-tumor tissues, and RNA modification in GBM. *RPP30* regulates protein expression in GBM by affecting post-transcriptional modification of proteins and functional accumulation of these proteins indicates that these proteins are mainly involved in the activation of cancer signaling pathways (10). In addition, downregulation of *RPP30* expression in human astrocyte (HA) cells promotes the proliferation of HA cells, while overexpression inhibits the activation of tumor-related pathways and the proliferation of HA cells, further confirming the close relationship between *RPP30* and the occurrence and development of GBM (10). Correlation analysis of *RPP30* expression levels with gene expression in cancer-related pathways, such as cancer, Wnt, and mitogen-activated protein kinase pathways in the Chinese Glioma Genome Atlas and The Cancer Genome Atlas databases show significant correlation (10). The Gene Expression Profiling Interactive Analysis (GEPIA2) database has been used to obtain broad knowledge of the relationship between *RPP30* (Ensembl ID: ENSG00000148688.13) and tumors (**Figure 2**) (8). *RPP30* expression was significantly different in tumor tissues (higher) and non-tumor tissues in diffuse large B-cell lymphoma, pancreatic adenocarcinoma (PAAD) and thymoma (THYM) (**Figure 3**). These data also show that there is no significant difference in *RPP30* expression levels in different stages of those tumors while high expression of *RPP30* is correlated with lower overall survival in PAAD using data from the GEPIA2 public database (http://gepia2.cancer-pku.cn/#index). *RPP30* gene expression is high under epidermal development, cell differentiation, and keratinocyte differentiation processes, which play important roles in the differentiation of gastric epithelial cells. Recently, Kan et al. used TGCA RNA-seq to explore the role of *RPP30* expression in gastric cancer. They found that *RPP30* protein expression was positively correlated with the number of T helper 2 cells, active dendritic cells, and T helper 1 cells, and negatively correlated with the number of T helper 17 cells. They also found that *RPP30* RNA expression in gastric cancer (GC) tissue is higher than that in normal tissue and higher *RPP30* RNA expression is related to worse overall survival (OS) at the T1, T2, and N0 stages of the tumor. The mechanism may be that *RPP30* RNA expression is upregulation *via* the G alpha S signaling pathway, neuronal system, and olfactory transduction, in addition to increasing cAMP levels, which are tightly correlated with GC histopathology. *RPP30* could regulate tRNA modification, transcriptional replication, DNA repair, replication fork stagnation, and protein expression, which are correlated with cancer cell proliferation (65, 66).

### Other diseases

Lee et al. Have found that rpp30 may be related to genetic factors of LBMD through genome-wide association studies involving two signaling pathways of eight related diseases (9). No further association between rpp30 and LBMD has been reported. *RPP30* is indirectly related to some diseases, including lung diseases and pulmonary hypertension, secondary to autoimmune diseases (62).

Currently, the research on genes and diseases is extremely in-depth and making rapid progress. Although rpp30 is involved in basic life activities, only a few human diseases have been confirmed to be related to rpp30, and even fewer have been confirmed to have abnormal expression. These results further reflect the stable expression of rpp30 and how well conserved it is.

## *RPP30* distribution and factors influencing *RPP30* expression

**Figure 4:**
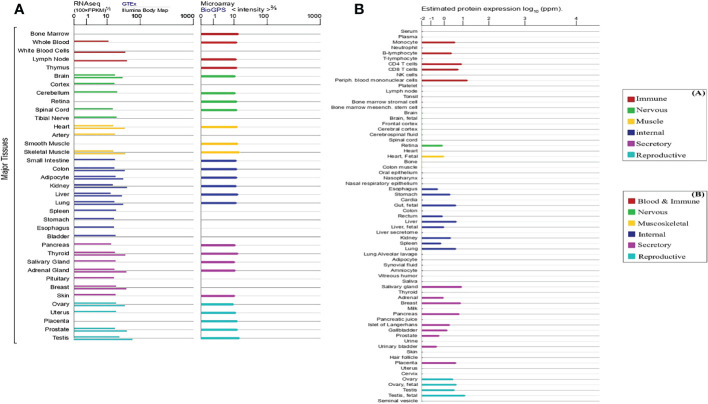
Expression level of human ribonuclease P protein subunit p30 (*RPP30*) RNA and protein in different tissues and cells (line colors indicate tissue types and length indicates levels of expression). **(A)** Expression of *RPP30* RNA using RNA sequencing (left: expression results above gray horizontal lines are from the Genotype-Tissue Expression (GTEx) database and those below are from the Illumina Body Map) and microarray (right: from the BioGPS database); **(B)** Protein expression in normal tissues and cell lines from Proteomics DB, the MaxQuantDataBase (MaxQB), and Multi-Omics Profiling Expression Database (MOPED).

Although *RPP30* is associated with appellate disease, the expression of *RPP30* in normal tissues and most tumor cells is stable. Approximately 3647 species have *RPP30* subunits and 424 organisms have orthologs of human *RPP30*. *RPP30* RNA is widely expressed in 27 human tissues, including testis, heart, kidney, lung, thymus, and lymph nodes, and more, among which testes and lymph nodes show the most expression and pancreas shows the least expression using data from the NCBI, InterPro (https://www.ebi.ac.uk/interpro/), and GeneCards public databases. In **Figure 4A**, although there are differences in *RPP30* RNA expression levels calculated by different databases, *RPP30* RNA expression levels of different organizations calculated by the same database are basically the same, which is consistent with the results obtained by Bgee involving gene expression data in animals. However, the expression of human RPP30 protein is not as stable as *RPP30* RNA. There are differences in the expression of RPP30 protein among different tissues or cells and the RPP30 protein has weak expression in some tissues or cells, such as lymph node, brain, spinal cord, ovary, bone, colon, and liver secretion (**Figure 4B**). There have also been no reports using RPP30 protein as an internal reference for western blotting. To summarize, the expression level of *RPP30* RNA in human tissue is relatively high and stable and is suitable to be used as an internal reference gene (11, 67, 68). Mouse *RPP30* RNA is widely expressed in the central nervous system, bladder, brain, liver, and testis, etc., with higher expression in the central nervous system and lower expression in the adrenal gland and stomach.

At present, there are relatively few reports on factors affecting *RPP30* expression levels. Li, Zhai (10) have found that *RPP30* expression is affected by age-related factors. Using analysis of age-related genes, *RPP30* expression was negatively correlated with increased age, indicating that the change in *RPP30* expression may be related to cell senescence. Li, Xiong (6) have found that *RPP30* expression is upregulated after rice has been infected with fungal and bacterial pathogens. Mattijssen, Welting (69) have speculated that the expression of housekeeping genes may be altered in the growth plates of patients with cartilage-hair hypoplasia.

## Application of *RPP30* as an internal reference gene

**Figure 5:**
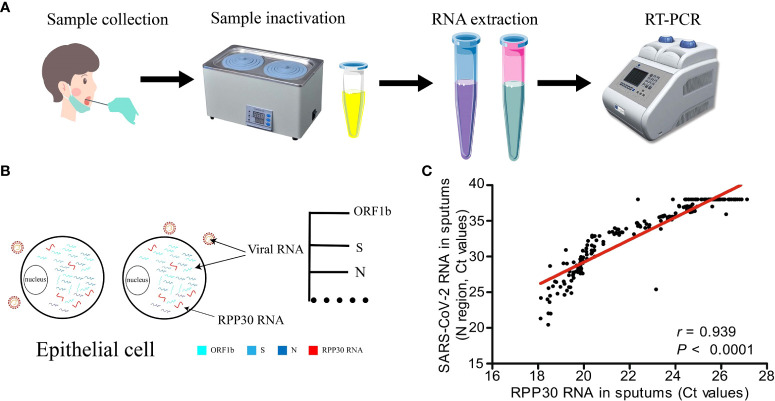
Ribonuclease P protein subunit p30 (*RPP30*) is used as an internal reference gene in the detection of SARS-CoV-2. **(A)** Sample collection and reverse transcription-polymerase chain reaction (RT-PCR), **(B)** Viral RNA coexists with *RPP30* in epithelial cells, **(C)** Detection results of *RPP30* and SARS-COV-2 are significantly positively correlated (11).

*RPP30* has been used as an internal reference gene in the detection of severe acute respiratory syndrome coronavirus 2 (SARS-CoV-2). Coronavirus disease 2019 (COVID-19) broke out in Wuhan, China in December 2019 and then spread widely around the world, with strong infectivity (70). Despite its reputation as the gold standard for the detection of SARS-CoV-2, RT-PCR often produces false negative results in detection and diagnosis (71). This may be related to sample quality or changes of primer/probe binding site sequences, but the latter is less likely (72, 73). *RPP30* is a single copy sequence gene stably expressed in the human genome, which has a good amplification efficiency, shows 100% sensitivity and specificity, and is not affected by swabs and methodology (74). Compared to other internal parameters, only *RPP30* exists in all types of SARS-COV-2 infection samples (67). **Figure 5A** is the flow of RT-PCR. Both *RPP30* RNA and viral RNA were present in epithelial cells (**Figure 5B**), and *RPP30* RNA levels were closely related to SARS-CoV-2 RNA levels in respiratory tract samples (**Figure 5C**). Thus, *RPP30* RNA may be used to control sample quality and the *RPP30* Ct cutoff value may effectively identify false negative results (11, 72), which may increase sensitivity and reduce the spread of SARS-CoV-2. In addition to evaluating the quality of the sample, *RPP30* may also determine whether mRNA has been extracted successfully and whether there is inhibition in the PCR (75).

*RPP30* is used as an internal reference gene to determine the best effective drug concentration for tumor treatment (76). The efficacy of traditional antineoplastic drugs is evaluated by calculating the lethality of drugs to all cells *in vitro*, so it is impossible to measure the lethality of antineoplastic drugs to normal cells, which is often accompanied by unpredictable side effects. Because *RPP30* is stably expressed in the vast majority of tumor cells and non-tumor cells, while the neurofibromatosis type 1 (NF1) gene loses heterozygosity in tumor cells, the number of tumor cells may be evaluated by the quantitative RT-PCR ratio of *NF1* to *RPP30*, which may be used to evaluate the efficacy and side effects of tumor drugs, and may also be used in personalized adjuvant chemotherapy. Due to the different behavior of cells *in vivo* and *in vitro*, this method has some limitations (76–78). As an internal reference gene, *RPP30* may also accurately and effectively evaluate the concentration of antiretroviral drugs in cells (79).

*RPP30* is used as an internal reference gene to analyze the feasibility of HIV DNA detection in cerebrospinal fluid (CSF) (80). *RPP30*, as a housekeeper gene, is highly conserved and widely expressed in human tissues and may be used as an internal reference gene to detect the number of leukocytes in CSF. Using droplet digital PCR (dd-PCR) detection, the level of HIV DNA in CSF cells is not correlated with *RPP30* levels, indicating that the detectability of HIV DNA does not depend entirely on the number of cells available in each sample. Then, the correlation between the level of HIV DNA in the CSF and the level of HIV RNA in peripheral blood cells, as well as the relationship between the virus inhibition and non-inhibition subgroups, may be analyzed to explore the detectability of HIV DNA level in CSF.

*RPP30* is used as an internal reference gene in diagnostic experiments (68). At least 5.5% of all pathogenic genetic changes in humans are large genome deletions or duplicates (81). With the discovery of disease-related genes, dd-PCR has been used to quantify the copy number of genes to diagnose diseases (82). Because of its conserved sequence and stable expression in almost all cells, *RPP30* is widely used as an internal reference gene (83). For example, *RPP30* has been used as an internal reference in real-time fluorescent PCR or dd-PCR to quantify the survival of motor neuron 1 gene, T-cell receptor excision circles, and Kappa-deleting recombination excision circles; to screen neonatal spinal muscular atrophy, severe combined immunodeficiency disease, and detect immune remodeling of the thymus and bone marrow (84–86); and to quantify the sex-determining region Y gene to detect male/female chimerism, which may track chimerism after hematopoietic stem cell transplantation (87). *RPP30* has also been used as an internal reference in single-cell dd-PCR to evaluate the genomic DNA of rare circulating fetal cells in peripheral blood samples of pregnant women with male fetuses and validate the concept of non-invasive prenatal diagnosis (88). There are also many reports on the use of *RPP30* as an internal reference in different molecular biology techniques for the diagnosis of human diseases, as shown in **Table 1**.

**Table 1:** Application of *RPP30* as an internal reference.

Molecular biology technology	Disease		Disease-related genes	Reference
Multiple Dd-PCR	Tumor disease	Ovarian cancer	*BRCA1*	(89)
Nanofluid digital PCR array		Lung cancer	*EGFR*	(90)
Real-time quantitative PCR		Breast cancer	*ERBB2*	(91)
Dd-PCR	Childhood disease	Severe combined immunodeficiency	*TREC*	(92)
Real-time fluorescence quantitative PCR		Spinal muscular atrophy	*SMN1*	(93)
Dd-PCR	Blood disease	α-thalassemia	Alpha globin gene	(94)
Dd-PCR	Inflammatory disease	Psoriasis, Chronic obstructive Pulmonary disease, Crohn’s disease and reproductive tract infections	β-defensin	(95)
Dd-PCR	Others	Pelizaeus-Merzbacher disease	*PLP1*	(96)
Dd-PCR		Hearing loss	*STRC*	(97)

Dyavar et al. (79) used human and rhesus macaque (RM) gDNA templates to quantitate *RPP30* copies, and found a low coefficient of variation and strong correlation between human and RM gDNA templates and the number of *RPP30* copies in intra-laboratory (R2 = 0.996, p < 0.001; R2 = 0.975, p < 0.001), inter-laboratory (R2 = 0.997, p < 0.001; R2 = 0.989, p < 0.001), and inter-operational (R2 = 0.994, p < 0.001; R2 = 0.986, p < 0.001) studies, which confirms the high accuracy and precision of the *RPP30* dd-PCR assay. In addition, Profaizer and Slev (86) observed that *RPP30* dd-PCR could detect 2 copies/µL of genes, which is more accurate than the previous 24 copies/µL for qPCR.

## Differences from other internal reference genes

Housekeeping genes are mainly involved in the maintenance of basic cell functions and are thought to be expressed in all cells (98), They are widely used as internal controls to standardize the expression of genes in western blotting, northern blotting, and RT-PCR. The ideal housekeeping gene should be expressed at the same level in all tissues (99). At present, frequently used housekeeping genes are *β-actin* and *GAPDH*, in which *β-actin* has a molecular weight of approximately 42–43 kDa and is composed of 375 amino acids. It is widely distributed in the cytoplasm and is involved in cell movement, structure, and integrity (100), whereas *GAPDH* is an enzyme with a molecular weight of approximately 37 kDa and is involved in glycolysis, DNA repair, tRNA output, membrane fusion, and transport (101). However, there are increasing reports that the RNA expression level of these genes is affected by the physiological/pathological state, experimental conditions, and tissue types (12–14). Thus, the factors affecting mRNA expression levels of *RPP30*, *GAPDH*, and *β-actin* were compared.

**Table 2** shows that the length of the *RPP30* amplification product is smaller than that of *β-actin* and *GAPDH*, which reduces errors and improves efficiency during the process of *RPP30* amplification. **Table 3** lists the pseudogenes found in *β-actin* and *GAPDH*, but, to date, no pseudogenes have been found in *RPP30*. The existence of pseudogenes reduces the amplification efficiency of genes and reduces the accuracy of their use as internal reference genes for standardization (122, 123). Therefore, using *RPP30* as the internal reference gene may be more accurate.

**Table 2:** Internal reference gene primer sequences.

Target gene	Forward primers	Reverse primers	Product length (bp)	Reference
*RPP30*	5′-GATTTGGACCTGCGAGCG-3′	5′-GCGGCTGTCTCCACAAGT-3′	62	(83, 96)
*β-actin*	5′-CAGACATCAGGGTGTGATGG-3′	5′-TCAGGGGCTACTCTCAGCTC-3′	183	(13)
*GAPDH*	5′-TGGGCAGATGCAGGTGCTGA-3′	5′-TGGTGCACGATGCATTGCTGAGA-3′	201	

**Table 3:** Comparing *RPP30* with other internal parameters.

Gene	The function of encoding proteins	Pseudogenes	Expression level of mRNA			
			Pathological conditions	Experimental conditions	Tissue types	Other
*RPP30*	Realize RNase P RNA binding activity and participate in the excision of tRNA 5-precursor	No found	DLBC↑, PAAD↑, THYM↑	Rice infection ↑ (6)	The expression between different tissues of human body is relatively stable	Age↓ (10)
*β-actin*	Participate in the movement, structure and integrity of cells	Exist (102)	Tumor↑ (103–105), Steatosis↓, Alcoholic hepatitis↑ (106), AD↓ (107)	Serum↑ (108), miR-644a↓ (109), Hypoxia↑ (110), HSV-1↓ (111), Exercise↑ (112), Fasting ↓ (113), Hyperglycemia↓ (114)	Unstable in different tissues of human body and at the stage of lymphocyte activation (115, 116)	Age↓ (117)
*GAPDH*	Involved in glycolysis, DNA repair, tRNA output, membrane fusion and transport	Exist (118)	Tumor↑ (103, 104), Steatosis ↓, Alcoholic hepatitis ↑ (106)	Serum↑ (108), miR-644a↓ (109), Insulin↑ (119), Hypoxia↑ (120), NO↑ (121)		Age↓ (117)

↑ indicates that the expression level is higher than that of the normal control in this cell, ↓ indicates that the expression level is lower than that of the normal control in this cell. AD, Alzheimer’s disease, HSV-1, herpes simplex virus, NO, nitric oxide.

Numerous reports suggest that gene expression levels of *β-actin* and *GAPDH* are affected by many factors under different pathological conditions, such as tumor cells and non-tumor cells (103–105, 124), steatosis and alcoholic hepatitis (106), and Alzheimer’s disease (107). Under different experimental conditions, expression levels of the traditional internal reference genes, *β-actin* and *GAPDH*, vary greatly, such as in serum-stimulated fibroblasts (108), miR-644a (109), dietary conditions (125), and other conditions (110–114, 119–121). In addition, *β-actin* has extensive variation in mouse lymphocytes and is not appropriate for use as an internal reference gene for the quantitative PCR analysis of mouse lymphocytes (126), since such changes may lead to data divergence and inaccuracy. At present, there are few reports on factors affecting *RPP30* expression levels, which may be related to the existence of *RPP30* in all three fields of life (archaea, bacteria, and eukaryotes), and because it is widely expressed in different tissues whose gene sequences are conserved and homologous, such as in humans, chimpanzees, rhesus monkeys, mice, fruit flies, *Saccharomyces cerevisiae*, and archaea (127). In addition, currently, research on *RPP30* is scant.

The mRNA of *β-actin* and *GAPDH* are not highly expressed in all cell types or tissues of chicken embryos, and the expression levels are different in different tissues (115), which is similar to the 15-fold difference between the highest and lowest expression levels of *GAPDH* in different human tissues observed by Barber et al. (128). *GAPDH* expression levels also vary in different varieties of the same plant (129). Furthermore, *β-actin* and *GAPDH* expression levels fluctuate significantly at different stages of lymphocyte activation (116), which may be related to their participation in other cellular biology functions. *RPP30* mRNA expression in different tissues is more stable than those of *β-actin* and *GAPDH*. In addition, *RPP30* is widely expressed in 27 human tissues, is relatively conserved in structure and function, is not correlated with DNA content in the sample, and is not affected by the content of genes to be tested, resulting in high application value in a series of samples with scarce and uneven DNA content (91). Currently, to reduce the inaccurate data caused by differences in the expression of internal reference genes among different tissue types, *RPP30* has become the main internal reference gene for quantitative detection of genes (130).

Moreover, the expression level of the three genes is affected by age (10, 117); their expression level decrease with age, but it is not known whether the specific mechanism is the same. There are also differences in the expression level of *β-actin* at different developmental stages (131). The factors affecting the expression level of *RPP30* RNA in different pathological states, experimental conditions, and tissue types is lower than that of the commonly used internal reference genes, *β-actin* and *GAPDH*. *RPP30* has good amplification efficiency and may be better used in RT-PCR experiments. Currently, there is no housekeeping gene that has stable expression, is abundant, and consistent under any condition (132). Therefore, specific reference genes should be verified and selected according to experimental conditions and sample type (102, 118).

## Conclusion

*RPP30* is a highly conserved gene that has homologous genes in 16 species. Although *RPP30* is a housekeeping gene and its encoding protein is a key subunit that maintains basic life activities, it is rarely reported to be associated with human diseases, and is overexpressed only in a few patients with cancer. In addition, compared to traditional reference genes, *RPP30* has advantages of short sequence length, is widely and uniformly expressed in various tissues, and its expression level is rarely disturbed by external factors. Overall, *RPP30* has great prospects and value as an internal reference gene. To date, *RPP30* has been used as an internal reference for nucleic acid tests of Sars-CoV-2, evaluation of therapeutic drugs and drug side effects, analysis of the feasibility of HIV detection, and many other diagnostic experiments. However, due to the unstable detection results of the *RPP30* protein, there are no studies that have used *RPP30* as a reference in western blotting.

In such conditions, *RPP30* may not be the first choice as a reference gene for these tests. Regardless of which kind of reference that is chosen, it may be affected by a few inevitable conditions. Thus, the correct reference to be used for these tests should be further explored.

## Author contributions

JW, SY and YW collected literature and wrote the original draft and prepared the figures and tables. JZ revised the original draft. ZZ conceived the idea and revised the manuscript. All authors contributed to the article and approved the submitted version.

## Funding

The study was supported by Anhui Provincial Natural Science Foundation (grant number 2108085MH298) and the Scientific research project of Anhui Medical University (grant number 2019GMFY02, 2021lcxk027). The funders had no role in the study design, data collection and analysis, decision to publish, or preparation of the manuscript.

## Acknowledgments

We would like to thank Editage (www.editage.cn) for English language editing.

## Conflict of interest

The authors declare that the research was conducted in the absence of any commercial or financial relationships that could be construed as a potential conflict of interest.

## Publisher’s note

All claims expressed in this article are solely those of the authors and do not necessarily represent those of their affiliated organizations, or those of the publisher, the editors and the reviewers. Any product that may be evaluated in this article, or claim that may be made by its manufacturer, is not guaranteed or endorsed by the publisher.
